# Mechanical ventilation as a major driver of COVID-19 hospitalization costs: a costing study in a German setting

**DOI:** 10.1186/s13561-023-00476-1

**Published:** 2024-01-16

**Authors:** Leslie R. Zwerwer, Jan Kloka, Simon van der Pol, Maarten J. Postma, Kai Zacharowski, Antoinette D. I. van Asselt, Benjamin Friedrichson

**Affiliations:** 1 Department of Health Sciences, University of Groningen, University Medical Center Groningen, Hanzeplein 1, 9713 GZ Groningen, The Netherlands; 2 Center for Information Technology, University of Groningen, Groningen, The Netherlands; 3 Department of Anaesthesiology, Intensive Care Medicine and Pain Therapy, University Hospital Frankfurt, Goethe University, Frankfurt, Germany; 4 Health-Ecore, Zeist, The Netherlands; 5 Department of Economics, Econometrics and Finance, Faculty of Economics and Business, University of Groningen, Groningen, The Netherlands; 6 Department of Epidemiology, University of Groningen, University Medical Center Groningen, Groningen, The Netherlands

**Keywords:** Covid-19, Hospitalization costs, Germany, DRG-system, Intensive care

## Abstract

**Background:**

While COVID-19 hospitalization costs are essential for policymakers to make informed health care resource decisions, little is known about these costs in western Europe. The aim of the current study is to analyze these costs for a German setting, track the development of these costs over time and analyze the daily costs.

**Methods:**

Administrative costing data was analyzed for 598 non-Intensive Care Unit (ICU) patients and 510 ICU patients diagnosed with COVID-19 at the Frankfurt University hospital. Descriptive statistics of total per patient hospitalization costs were obtained and assessed over time. Propensity scores were estimated for length of stay (LOS) at the general ward and mechanical ventilation (MV) duration, using covariate balancing propensity score for continuous treatment. Costs for each additional day in the general ward and each additional day in the ICU with and without MV were estimated by regressing the total hospitalization costs on the LOS and the presence or absence of several treatments using generalized linear models, while controlling for patient characteristics, comorbidities, and complications.

**Results:**

Median total per patient hospitalization costs were €3,010 (Q1 – Q3: €2,224—€5,273), €5,887 (Q1 – Q3: €3,054—€10,879) and €21,536 (Q1 – Q3: €7,504—€43,480), respectively, for non-ICU patients, non-MV and MV ICU patients. Total per patient hospitalization costs for non-ICU patients showed a slight increase over time, while total per patient hospitalization costs for ICU patients decreased over time. Each additional day in the general ward for non-ICU COVID-19 patients costed €463.66 (SE: 15.89). Costs for each additional day in the general ward and ICU without and with mechanical ventilation for ICU patients were estimated at €414.20 (SE: 22.17), €927.45 (SE: 45.52) and €2,224.84 (SE: 70.24).

**Conclusions:**

This is, to our knowledge, the first study examining the costs of COVID-19 hospitalizations in Germany. Estimated costs were overall in agreement with costs found in literature for non-COVID-19 patients, except for higher estimated costs for mechanical ventilation. These estimated costs can potentially improve the precision of COVID-19 cost effectiveness studies in Germany and will thereby allow health care policymakers to provide better informed health care resource decisions in the future.

## Background

In December 2019, the first COVID-19 cases emerged in Wuhan, China [1]. The virus quickly spread around the rest of the world, causing intensive care units (ICU) globally to be overflooded [2–4]. To reduce transmission of the COVID-19 virus, governments across the globe posed several public health and social restrictions [5, 6]. During this pandemic, healthcare expenditures rose considerably. For instance, in Germany, healthcare expenditure increased from 2019 to 2020 with 1%, compared to an average annual increase of 0.05% in the decade before that [7]. Given that over 561,000 German citizens with COVID-19 were hospitalized during the first two years of the pandemic it is reasonable to assume that COVID-19 had a considerable effect on these healthycare expenditures [8, 9]. 

Many hospitalized COVID-19 patients develop severe complications, such as sepsis, acute respiratory distress syndrome and acute kidney injury and are therefore often in need of costly intensive treatments, e.g., mechanical ventilation or kidney replacement therapy [10–12]. Given the substantial number of hospitalized COVID-19 patients as well as the intensive treatment needed for these patients, the costs of hospitalized COVID-19 patients are expected to be considerable [13]. However, little is known about the exact costs of treating for these patients [14–17]. Many researchers use relatively crude estimated average hospitalization costs or costs for related diseases as a proxy for the actual costs of treating COVID-19 patients [13, 18–21].

To date, as far as we are aware, no study examined the costs of hospitalized COVID-19 patients in Germany. Average ICU costs for non-COVID-19 patients have been widely studied, however the costs of an ICU stay varies substantially between studies. For instance, in a multicentre study involving 222 German ICUs, a day on the ICU was valued at € 744 on average (inflated to 2021 using harmonised indices of consumer prices from Eurostat [22], rounded to whole euros) [23]. Tan et al. (2012) found an average of €1,462 per day (inflated to 2021 [22], rounded to whole euros) using a standardized costing methodology in a single German hospital [24]. Comparable costs were found by Martin et al. (2008), who estimated the costs of a day on the ICU in Germany using data from a single ICU to be on average € 1,434 and € 1,786 (both inflated to 2021 [22], rounded to whole euros), respectively without and with mechanical ventilation [25]. In a study involving 51 German ICUs much lower daily costs were found, that is € 901 and € 1,254 (inflated to 2021 [22], rounded to whole euros), respectively without and with mechanical ventilation [26]. Other researchers estimated average daily ICU costs in Germany ranging from € 1,179—€ 1,280 (inflated to 2021 [22], rounded to whole euros) [27, 28]. One study assessed the total per patient hospitalization costs for patients with influenza in Germany, which may be most comparable to COVID-19. In a nationwide inpatient sample including non-ICU (93.9%) and ICU (6.1%) patients, the total median costs per patient and per admission were € 1,858 (inflated to 2021 [22], rounded to whole euros) [29].

While literature is available for costs of hospitalized non-COVID-19 patients in Germany, the question remains if these costs can be used as a proxy for the costs of hospitalized COVID-19 patients. Furthermore, it is yet to be determined if these costs are appropriate throughout the COVID-19 pandemic. Since the beginning of the pandemic the treatment of COVID-19 patients evolved heavily. Over time treatments for hospitalized COVID-19 patients became more effective. Besides, several new mutations, such as Delta and Omicron emerged, which led to changes in the clinical picture. For example, patients infected with the Delta variant were much more likely to be admitted to the ICU compared to patients infected with the Alpha variant [30]. Finally, currently we are moving from a pandemic to an endemic situation [31]. All these changes potentially influence the costs for hospitalized COVID-19 patients.

At the same time, accurate estimations of the expenditures for treating COVID-19 patients are essential to allow policymakers to make informed health care resource decisions [14, 15, 32, 33]. Currently, as we are moving into the endemic situation, accurate estimations of these expenditures are more important than ever as decision makers have to make many health economic decisions regarding endemic COVID-19. In this retrospective study, we will therefore analyze the costs for German COVID-19 hospitalizations from the beginning of the pandemic until mid-2021 using a top-down approach. In the following sections we will discuss from a German statutory health insurance perspective the following research questions: 1). What are the total per patient COVID-19 hospitalization costs for German non-ICU and ICU patients? 2).How do the total per patient COVID-19 hospitalization costs for German non-ICU and ICU patients develop over time between the beginning of the pandemic and mid-2021? and 3). What are the daily COVID-19 hospitalization costs for non-ICU and ICU patients in Germany?

## Methods

### Data collection

In 2003, the German diagnosis related groups (DRG) system has been introduced, which is a standardized case-based reimbursement system based on diagnoses. For the reimbursement of treatments, all hospitals must provide information based on the International Statistical Classification of Diseases and Related Health Problems edition 10 (ICD-10) and the Operation and Procedure Classification System version 2020. To further develop the DRG system, all hospitals in Germany are obliged under §21 of the Hospital Finance Act (KHG) to forward these data anonymously to the Institute for the Hospital Remuneration System (InEK). For this study, these anonymized data were used. The study was conducted in accordance with the Declaration of Helsinki and ethical approval was provided by the Ethical Committee of the University Hospital Frankfurt (Chair: Prof. Dr. Harder, Ref: 2021–36). Moreover, the study generally followed the checklist for Development and Assessment of Cost-of-Illness Studies from Müller et al. (2017), which provides a framework for costing studies adapted to the German situation [34]. All inpatients with a positive SARS-CoV-2 Reverse transcription Polymerase chain reaction (rt-PCR) smear admitted between February 1, 2020 and July 1, 2021 to the University Hospital Frankfurt am Main were included in this study. Most of these patients had COVID-19 as their primary diagnosis. There were no missing data. The total per patient hospitalization costs, overall length of stay (LOS), general ward LOS, ICU LOS, duration of mechanical ventilation and several other treatments, such as duration of extracorporeal membrane oxygenation (ECMO) were recorded for all patients. The total per patient hospitalization costs used in this study were non-negotiable DRG reimbursement fees and would therefore have been similar in all other German hospitals for these same patients. Total per patient hospitalization costs included all hospitalization costs except extrabudgetary compensations (i.e. “Zusatzentgelt”), such as educational infrastructure costs and extrabudgetary compensation for ECMO or dialysis, which are subject to negotiation and as such not disclosed.

### Pre-processing and descriptive analysis

First, all subjects below the age of 18 years were removed from the data. These patients were excluded from the analysis as clinically speaking they form a different population and receive a materially different treatment for COVID-19 (e.g., paediatric ICU). The sample was split into two parts based on the type of patient: non-ICU or ICU. Non-ICU patients were admitted to the general ward only, ICU patients were admitted to the ICU but could also be in the general ward for part of their hospital stay. Both samples were considered separately for analysis. The Elixhauser comorbidity score was used to examine the comorbidity of patients [35]. The Elixhauser comorbidity score, is based on secondary ICD-10 codes and reflects the pre-existing comorbidities in patients. It is a well-established score for risk adjustments. In addition, this score can be used to predict hospital mortality, adverse events, LOS, and hospital discharges [35, 36].

Descriptive analyses were performed for both samples. The number of non-ICU and ICU patients admissions were visualized. Changes in patient characteristics, general ward LOS, ICU LOS and duration of mechanical ventilation over time (per quarter) were captured visually.

### Total per patient hospitalization costs for non-ICU and ICU COVID-19 patients

Descriptive analyses were performed on the total per patient hospitalization costs for non-ICU and ICU patients separately. Total per patient hospitalization costs over time were visualized per quarter and year. Linear trends in the total per patient hospitalization costs over time were captured visually.

### Daily hospitalization costs of non-ICU and ICU COVID-19 patients

#### Generalized additive modelling

To estimate the costs for each additional day in the hospital for non-ICU patients and ICU patients we used generalized linear models (GLM). A GLM is a flexible form of an Ordinary Least Square regression as it allows for a link function, which can transform the dependent variable. In addition, different families of error distributions can be used. Consequently, depending on the family of the error distribution, the variance can mathematically depend differently on the mean value.

Costs for non-ICU COVID-19 patients were estimated using the subsample of the non-ICU COVID-19 patients. The total per patient hospitalization costs were regressed on the LOS at the general ward and the presence or absence of dialysis using GLM, while controlling for patient characteristics, the Elixhauser comorbidity score and complications. Complications included myocardial infarction, stroke, intra cerebral bleeding and embolisms, such as pulmonary embolisms and thromboembolisms. The subsample with the ICU patients was used to estimate the costs for the ICU patients. The costs were estimated by regressing the total per patient hospitalization costs on the general ward LOS, the non-mechanically ventilated ICU LOS, and the duration of mechanical ventilation as well as the presence or absence of several other treatments, using GLMs, while controlling for patient characteristics, complications and the Elixhauser comorbidity score.

Models were estimated with different error distribution families: Gaussian, Gamma and inverse Gaussian distribution. All models were fitted with the identity link function and the log link function. Both functions were considered plausible candidates for the link function. We hypothesised that costs, excluding the first few days in the hospital, act additively, which would argue for an identity link function. However, considering the non-negativity of the costs data the log link function would be a good second candidate.

#### Propensity score model

All models used for estimating the costs of an additional day in the hospital were fitted doubly robust [37], meaning that potential confounders were controlled for and a continuous propensity score weighting was applied. Unlike controlling for covariates in regression or propensity score weighting a doubly robust method assures unbiased estimates when one of the aforementioned models is mis specified. Propensity scores were estimated for the non-ICU patients and the ICU patients separately using covariate balancing propensity score (CBPS) for continuous treatment. In CBPS covariates are balanced by mathematically minimizing the differences in the means and standard deviations between the control and treatment group [38]. The extension of the CBPS for continuous treatment is based on a similar mechanism. However, instead of minimizing means and standard deviations between the treatment and control group the covariance between the treatment and the covariates is minimized [39]. The general ward LOS and duration of mechanical ventilation was taken as the continuous treatment assignment in this study for respectively non-ICU and ICU patients. Hence, all baseline covariates were balanced in such a way that non-ICU patients with a short LOS in the general ward had similar baseline covariates compared to patients with a long general ward LOS. For the ICU sample, baseline covariates were balanced for the duration of mechanical ventilation. All pre-treatment covariates that were unbalanced were included in the CBPS to be balanced. Covariates were considered balanced whenever the adjusted correlation was lower than 0.1 [40] and the adjusted distributional balance in the scatterplots and histograms from the R package cobalt appeared balanced [41].

#### Model fit and assumptions

Model fit was compared using the Akaike Information Criterion (AIC) and the Bayesian information Criterion (BIC). Moreover, model assumptions were checked using the R package DHARMa [42]. DHARMa uses simulations to create interpretable standardized residuals from a fitted GLM model. These simulated residuals are compared to the residuals of the fitted model. To evaluate the fit and model assumptions of each fitted model we simulated 1,000 residuals and assessed the quantile–quantile (q-q) plots visually. Moreover, a Kolmogorov–Smirnov test was carried out and a dispersion test was performed to check for over or underdispersion. We used the outlier test in DHARMa to check if any of the residuals were significantly different from the simulated residuals. Based on the results on the aforementioned tests, the best fitted GLM family and link function were selected. Moreover, we checked for multicollinearity using the variance inflation factor (VIF). Finally, a sensitivity analysis was performed by deleting all outliers, as indicated by the DHARMa package, and refitting the model. An observation is flagged as an outlier by the DHARMa package if the observation is larger or smaller than all values simulated by the fitted model.

All analyses were performed in R version 4.0.3 [43] using libraries dplyr [44], ggplot2 [45], comorbidity [46], regclass [47], CBPS [48], cobalt [41] and DHARMa [42].

## Results

### Demographics

#### Admissions

**Fig. 1:**
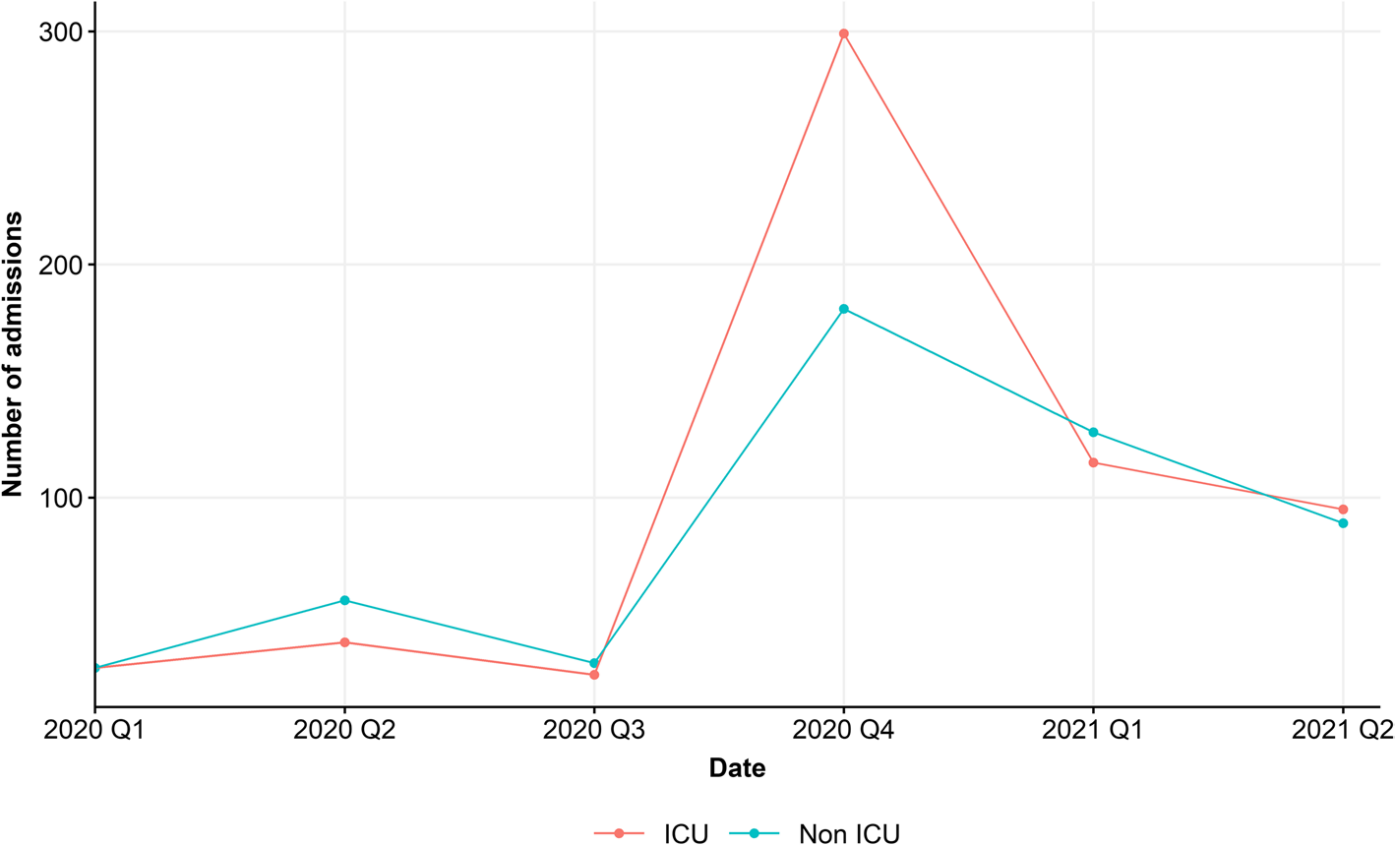
Number of non-ICU and ICU admissions over time

A total of 1,156 inpatients with a positive SARS-CoV-2 rt-PCR smear were included in the study. After removal of all patients below the age of 18 years the data consisted out of 1,108 patients. More specifically, there were 598 non-ICU patients and 510 ICU patients. The majority of the patients was admitted between February 1, 2020 and December 31, 2020, that is, a total of 681 hospital admissions. In 2020, 388 non-ICU patients were admitted to the general ward and 293 patients were admitted to the ICU. In the first half of 2021, 427 patients were admitted to the hospital, of which 210 non-ICU admissions and 217 ICU admissions. A more detailed overview of the hospital admissions can be found in Fig. 1, which shows the number of non-ICU and ICU admissions per quarter.

#### Basic demographics

Basic demographics of the non-ICU patients, the non-mechanically ventilated and mechanically ventilated ICU patients can be found in Table 1. Overall, patients in the ICU without and with mechanical ventilation were older compared to the non-ICU patients. The majority of patients was male, which was more pronounced in the ICU. Moreover, the Elixhauser comorbidity scores were higher for ICU patients without and with mechanical ventilation compared to non-ICU patients. Overall, most comorbidities like obesity, diabetes, congestive heart failure and chronic pulmonary disease were more common in the ICU. However, cancers were much less frequent in the ICU with mechanical ventilation. This difference was found to be significant.

**Table 1:** Demographics of the sample

Characteristics	Total (*N* = 1108)	General ward (*N* = 598)	ICU non-mechanical ventilation (*N* = 124)	ICU mechanical ventilation (*N* = 386)
Age, mean (sd)	60.35 (17.42)	58.24 (18.91)	62.21 (18.92)	63.01 (13.73)
Gender, % male	63.90	55.52	65.32	76.42
Elixhauser comorbidity score, mean (sd)	1.91 (1.87)	1.00 (1.21)	2.40 (1.89)	3.17 (1.91)
Obese (body mass index > 30, %)	^b^	≤ 1.67^a^	8.87	11.66
Hypertension (%)	34.57	21.24	49.19	50.52
Diabetic (%)	23.83	16.05	24.19	35.75
Chronic pulmonary disease (%)	7.49	4.52	12.90	10.36
Congestive heart failure (%)	6.32	2.17	10.48	11.40
Cardiac arrhythmias (%)	13.09	7.53	16.94	20.47
Valvular disease (%)	2.26	≤ 1.67^a^	≤ 8.06^a^	2.85
Peripheral vascular disorder (%)	3.43	≤ 1.67^a^	≤ 8.06^a^	6.48
Liver disease (%)	^b^	2.84	≤ 8.06^a^	10.88
Aids/HIV (%)	1.71	2.34	≤ 8.06^a^	≤ 2.59^a^
Cancers and lymphoma (%)	^b^	7.86	≤ 8.06^a^	3.63
Coagulopathy (%)	^b^	2.68	≤ 8.06^a^	32.64
Renal failure (%)	8.39	7.19	12.10	9.07

*ICU* Intensive Care Unit, *N* Number of subjects, *Sd* Standard deviation

^a^Ten or less patients, censored for privacy

^b^Omitted for privacy, preventing the observations flagged with ^a^ to be deducted from all the other columns

#### Demographics over time for non-ICU patients

**Fig. 2:**
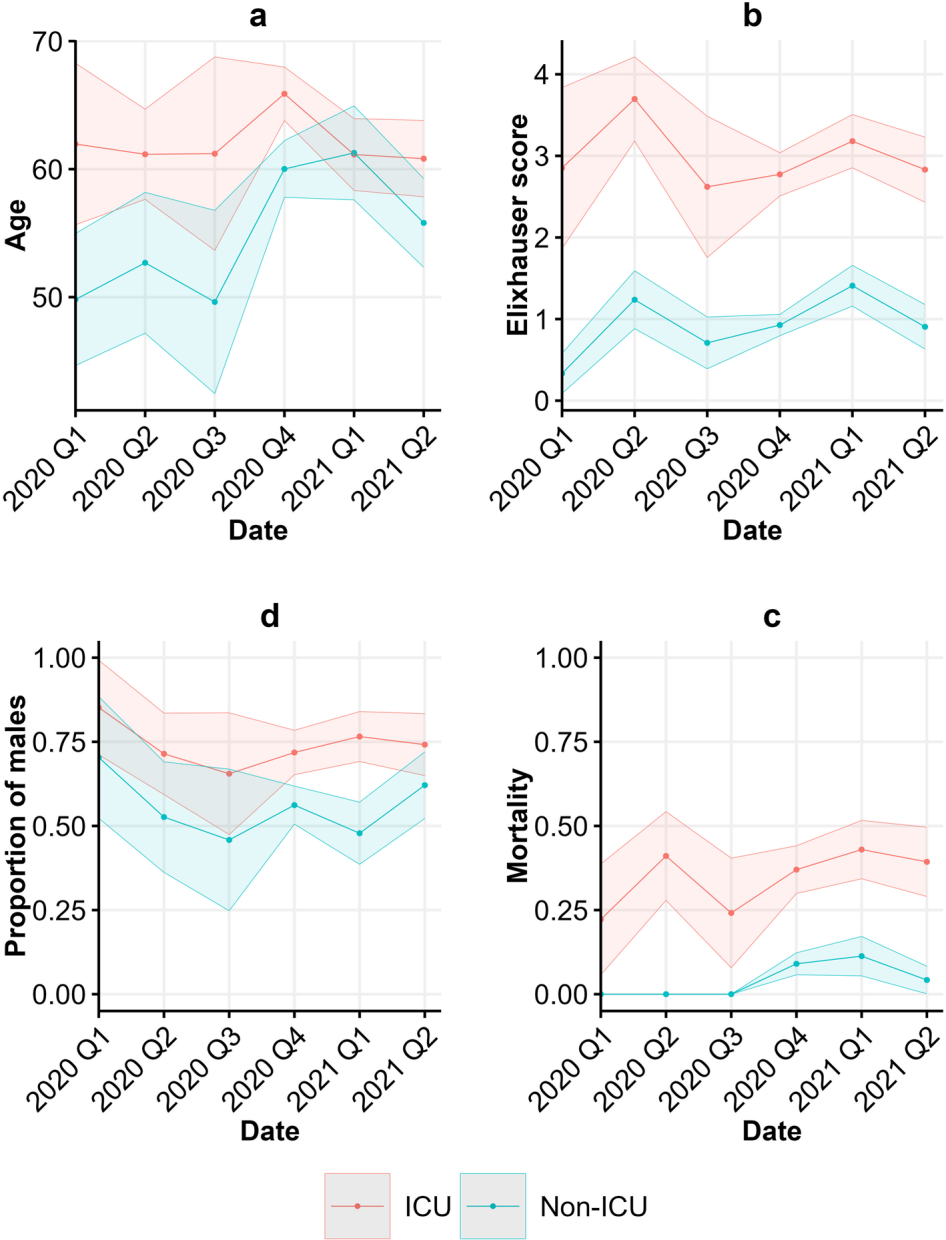
Observed mean and confidence intervals (CIs) of (**a**) age, (**b**) Elixhauser comorbidity score, (**c**) proportion of males, and (**d**) average mortalities per quarter for non-ICU patients (blue) and ICU patients (red)

Figure 2 shows the variation in the average age, average Elixhauser comorbidity score, the proportion of males and the mortality over time for non-ICU and ICU patients. The average age of non-ICU COVID-19 patients showed considerable fluctuation over time. These plots show that non-ICU patients were on average older in the fourth quarter of 2020 and the first quarter of 2021. A possible explanation for this could be that up until the third quarter of 2020 there were still few COVID-19 cases at University Hospital Frankfurt am Main (see Fig. 1) and in the rest of Germany as well [9]. Therefore, at the beginning of the pandemic, many patients, especially younger ones, were prophylactically admitted to the hospital. However, starting in the fourth quarter of 2020, there was a massive increase in the number of COVID-19 patients and there was a shift towards older and more vulnerable non-ICU patients. Very young patients with mild symptoms were more likely to receive outpatient care due to the strain on hospitals.

The Elixhauser comorbidity score and proportion of males for non-ICU patients were relatively stable over time. However, non-ICU patients in the beginning of the COVID-19 pandemic had slightly lower Elixhauser comorbidity scores and were mainly male. The average mortality in the first three quarters of 2020 was zero for non-ICU patients.

#### Demographics over time for ICU patients

In the ICU, the average age was the highest for patients admitted in the fourth quarter of 2020. ICU patients with on average the highest and lowest Elixhauser comorbidity score were admitted in respectively the second and third quarter of 2020. In the beginning of the pandemic the vast majority of the patients admitted to the ICU was male. Over time this proportion decreased and stabilized around 71%. Finally, mortality was relatively stable over time, fluctuating around an average of 38%.

#### Treatments and complications for non-ICU and ICU patients

Table 2 shows the median LOS on the general ward and the ICU, the presence or absence of complications, the duration of mechanical ventilation and the duration and or presence/absence of other treatments.

**Table 2:** LOS in hours and days (rounded to integers), treatments and complications for the full sample, non-ICU patients, non-mechanically ventilated ICU patients and mechanically ventilated ICU patients

	Full sample (*N* = 1,108)	Non-ICU patients (*N* = 598)	Non-mechanically ventilated ICU patients (*N* = 124)	Mechanically ventilated ICU patients (*N* = 386)
Total costs per patient in euros (mean, median, [Q1-Q3])	14,655.05, 5,103.05 [2,686.20; 12,076.57]	4036.39, 3,009.75 [2,223.93; 5,272.72]	8118.03, 5,887.36 [3,053.67; 10,879.12]	33,205.70, 21,535.83 [7,503.64; 43,480.46]
Total LOS per patient in hours/days (mean, median, [Q1-Q3])	319, 216 [120;384], 13, 9 [5;16]	195, 168 [72;240], 8, 7 [3;10]	373, 264 [144;516], 16, 11 [6;22]	492, 360 [216;648], 21, 15 [9;27]
General ward LOS in hours/days (mean, median, [Q1-Q3])	173, 120 [24;219], 7, 5 [1;9]	195, 168 [72;240], 8, 7 [3;10]	297, 178 [74;390], 12, 7 [3;16]	100, 12 [1;133], 4, 1 [0;6]
ICU LOS in hours/days (mean, median, [Q1-Q3])	146, 0 [0;167], 6, 0 [0;7]	NA	76, 50 [24;95], 3, 2 [1;4]	394, 269 [135;532], 16, 11 [6;22]
Non mechanical ventilation ICU stay in hours/days (mean, median, [Q1-Q3])	53, 0 [0;59], 2, 0 [0;2]	NA	76, 50 [24;95], 3, 2 [1;4]	127, 76 [11;184], 5, 3 [0;8]
Duration of mechanical ventilation in hours/days (mean, median, [Q1-Q3])	93, 0 [0;12], 4, 0 [0;1]	NA	NA	267, 135 [10;375], 11, 6 [0;16]
ECMO duration in hours/days (mean, median, [Q1-Q3])	24, 0 [0;0], 1, 0 [0;0]	NA	NA	1654, 0 [0;0], 69, 0 [0;0]
Dialysis (%)	6.23	≤ 1.67^a^	≤ 8.06^a^	15.03
Cardiopulmonary resuscitation (%)	2.08	≤ 1.67^a^	≤ 8.06^a^	4.66
Myocardial infarction (%)	0.99	≤ 1.67^a^	≤ 8.06^a^	≤ 2.59^a^
Stroke (%)	≤ 0.90^a^	≤ 1.67^a^	≤ 8.06^a^	≤ 2.59^a^
Pulmonary embolism (%)	≤ 0.90^a^	≤ 1.67^a^	≤ 8.06^a^	≤ 2.59^a^
Intra cerebral bleeding (%)	≤ 0.90^a^	≤ 1.67^a^	≤ 8.06^a^	≤ 2.59^a^
Embolism/thrombosis (%)	≤ 0.90^a^	≤ 1.67^a^	≤ 8.06^a^	≤ 2.59^a^
Mortality (%)	21.39	7.36	12.10	46.11

*ECMO* extracorporeal membrane oxygenation, *ICU* Intensive Care Unit, *N* number of subjects, *LOS* length of stay, *Q1* first quartile, *Q3* third quartile

^a^Ten or less patients, censored for privacy

**Fig. 3:**
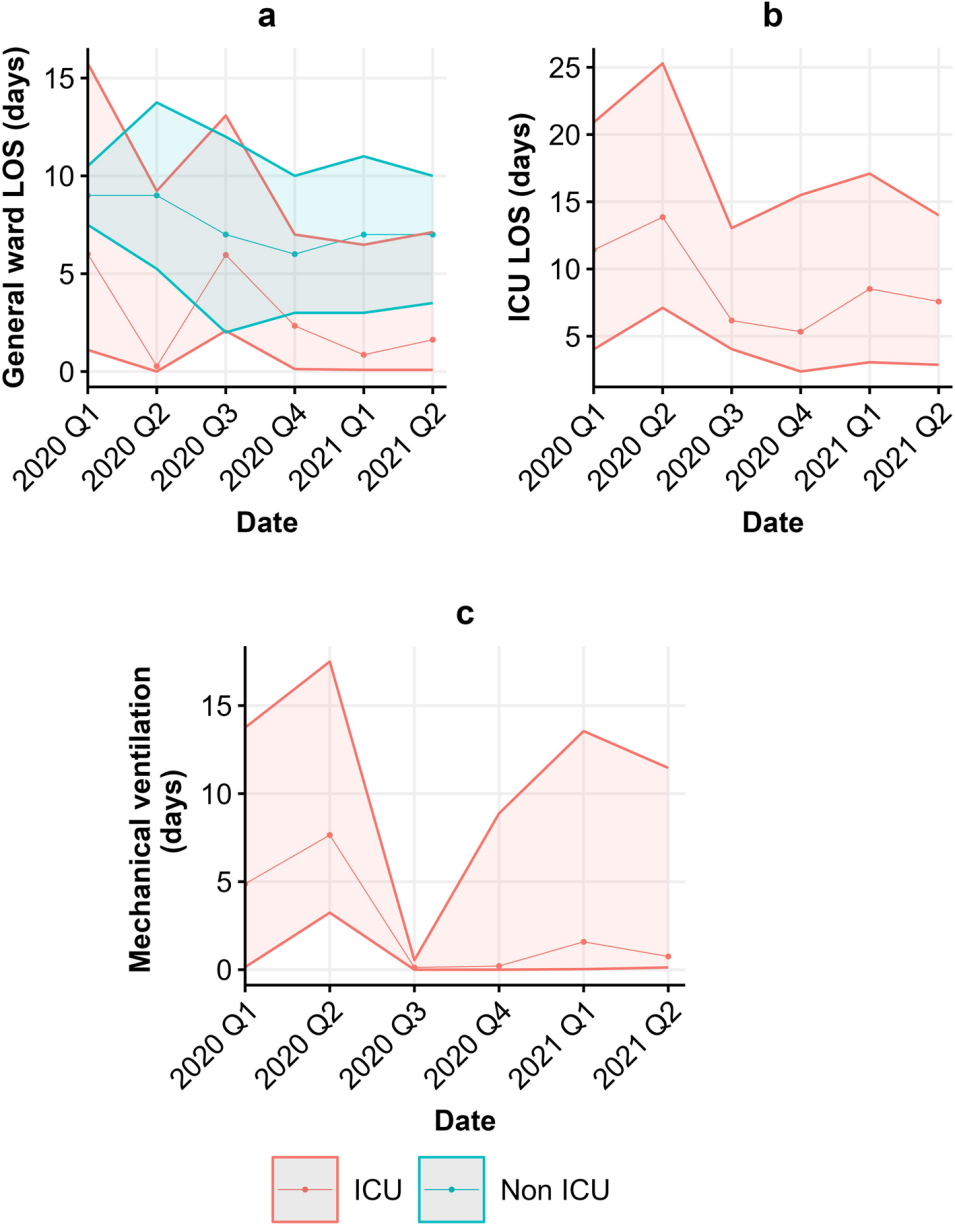
Observed median (Q1-Q3) of (**a**) general ward LOS, (**b**) ICU LOS, and (**c**) duration of mechanical ventilation over time for non-ICU (blue) and ICU patients (red). Note: ICU LOS and duration of mechanical ventilation were not applicable to non-ICU patients

In Fig. 3 we illustrate the median general ward LOS, median ICU LOS, and median duration of mechanical ventilation over time for both the non-ICU patients as well as the ICU patients. Compared to ICU patients, non-ICU patients had a longer LOS on the general ward. Overall general ward LOS decreased over time for non-ICU patients, while for the ICU patients general ward LOS varied over time with peaks at the first and third quarter of 2020. Remarkably, median general ward LOS for ICU patients in the second quarter of 2020 was less than a day (6.5 h). ICU LOS and duration of mechanical ventilation over time showed a similar pattern compared to the total per patient hospitalization costs of ICU patients. The ICU LOS and duration of mechanical ventilation peaked in the first two quarters of 2020 and had a slightly lower peak in the first quarter of 2021.

### Total per patient hospitalization costs for non-ICU and ICU patients

Total per patient hospitalization costs per patient for the full sample, that is non-ICU patients and ICU patients, ranged between € 684 and € 209,814 with a median of € 5,103 (Q1 – Q3: € 2,686—€ 12,077). More specifically, median total per patient hospitalization costs per non-ICU patients and per ICU patients, without and with mechanical ventilation were respectively € 3,010 (range: € 684—€ 45,803, Q1 – Q3: € 2,224—€ 5,273), € 5,887 (range: € 705—€ 38,028,Q1– Q3: € 3,054—€ 10,879) and € 21,536 (range: € 724—€ 209,814, Q1 – Q3: € 7,504—€ 43,480).

**Fig. 4:**
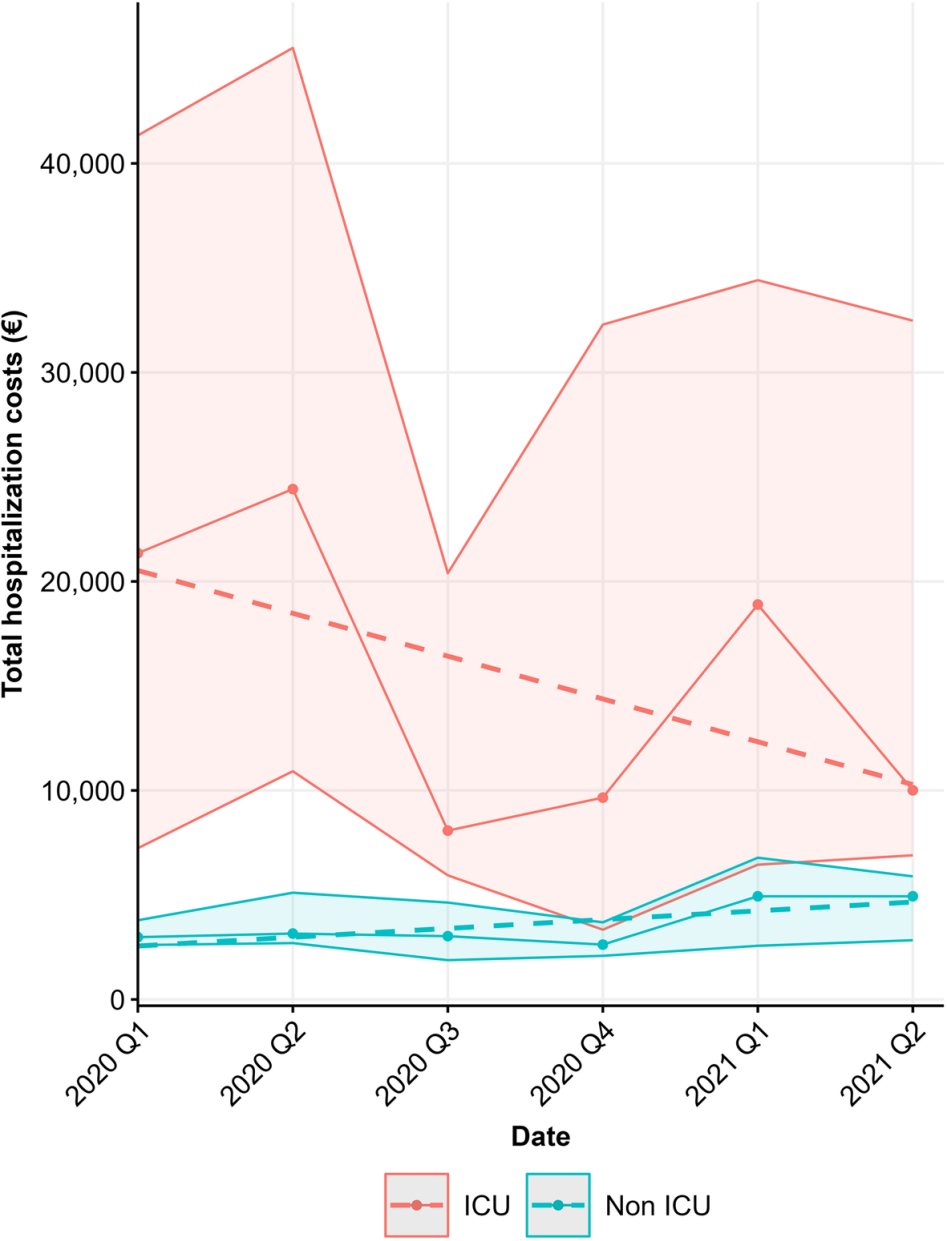
Observed median (Q1 - Q3) of total per patient hospitalization costs for non-ICU patients (blue) and ICU patients (red) fitted with a linear line

In Fig. 4 we illustrate the median total per patient hospitalization costs per patient over time for both the non-ICU patients as well as the ICU patients together with a fitted linear line. Total per patient hospitalization costs for non-ICU patients were relatively stable over time. There was a slight positive trend. Total per patient hospitalization costs for ICU patients were higher in the first half year of 2020 and the first quarter of 2021. The linear line showed a negative slope.

### Daily hospitalization costs for non-ICU patients

Age and Elixhauser comorbidity score were unbalanced across the LOS in the general ward for non-ICU patients and were therefore included in the CBPS. All these covariates were balanced after applying the propensity score weights created by CBPS. The effective sample size after adjusting for covariate unbalance using the estimated sample weights was 415.69.

**Fig. 5:**
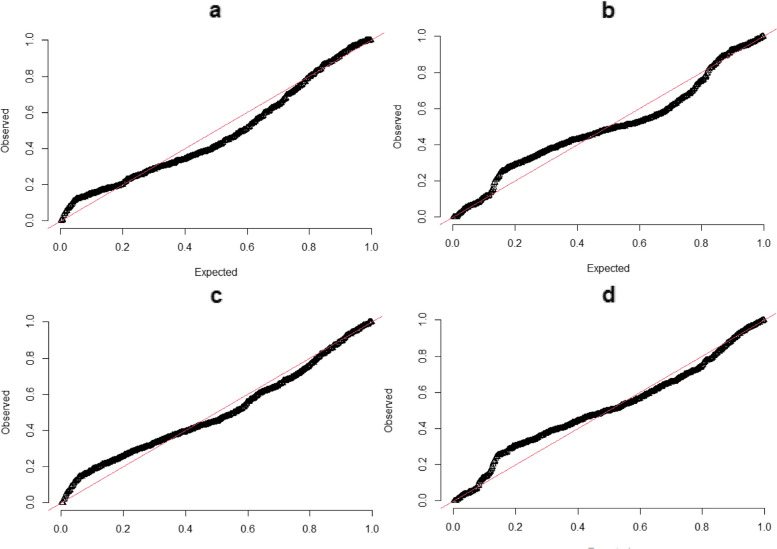
Q-q plots for non-ICU patients as generated by DHARMa with **a** Gamma error distribution with identity link function. **b** Gamma error distribution with log link function. **c** Inverse Gaussian error distribution with identity link function. **d** Inverse Gaussian error distribution with log link function. The best fit was provided by the GLM with an inverse Gaussian distribution with identity link function

We explored Gaussian, inverse Gaussian and Gamma distributions each with log and identity link. Considering AIC and BIC (Table 5 in Appendix), quantile–quantile plots and Kolmogorov–Smirnov test (Fig. 5 in Appendix and Table 6 in Appendix) we decided for an inverse-Gaussian distribution with identity link when modelling costs for non-ICU patients. The estimated coefficients for this GLM can be found in Table 3. Mortalities, higher Elixhauser comorbidity scores and longer LOS on the general ward were significantly positively associated with the total per patient hospitalization costs. Age and cardiopulmonary resuscitation were significantly negatively associated with the total per patient hospitalization costs. The estimated coefficients were not affected by multicollinearity.

**Table 3:** Estimated coefficients of GLM with inverse Gaussian error distribution and identity link function for non-ICU patients

Coefficient (standard error)	Full sample	Outliers removed
Constant	726.02 (95.58)***	622.49 (85.34)***
Age	-3.42 (1.50)*	-2.32 (1.33)
Gender female	74.70 (51.60)	87.10 (46.79)
Mortality	329.81 (104.27)**	93.55 (85.23)
Elixhauser comorbidity score	101.98 (22.64)***	79.95 (20.39)***
General ward (days)	463.66 (15.89)***	479.21 (14.76)***
Dialysis	72.49 (487.88)	61.78 (388.05)
Cardiopulmonary resuscitation	-639.31 (122.90)***	-377.82 (104.25)***
Complications	886.21 (852.03)	857.48 (773.09)
Number of samples	598	594
Weighted samples size	415.69	387.88
*P* value Kolmogorov–Smirnov test	< 0.001***	< 0.001***
*P* value dispersion test	< 0.001***	< 0.001***
*P* value outlier test	0.03*	1.00

^*^ *p* < 0.05; **p < 0.01; ***p < 0.001

In addition, sensitivity analysis was performed by deleting all outliers as indicated by the DHARMA package. None of the estimated coefficients changed majorly. However, age and mortality were no longer significant. The estimated GLM without outliers can be found in Table 3.

### Daily hospitalization costs for ICU patients

Age, gender and Elixhauser comorbidity score were unbalanced across the LOS in the ICU. All these covariates were balanced after applying the propensity score weights created by CBPS. The effective sample size after adjusting for covariate unbalance using the estimated sample weights was 350.57.

**Fig. 6:**
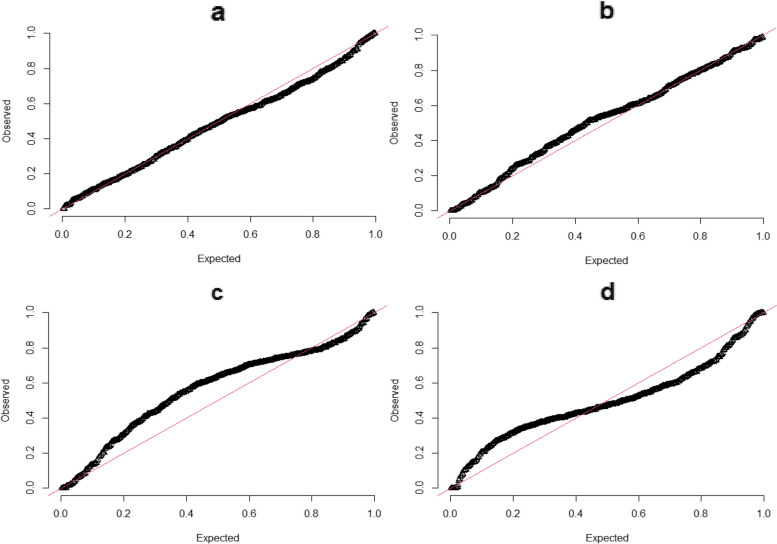
Q-q plots for ICU patients as generated by DHARMa with **a** Gamma error distribution with identity link function. **b** Gamma error distribution with log link function. **c** Inverse Gaussian error distribution with identity link function. **d** Inverse Gaussian error distribution with log link function. The best fit was provided by a GLM with a gamma error distribution and an identity link function

The Gaussian, inverse Gaussian and Gamma distributions each with log and identity link were explored as potential error distributions of the GLM. Considering AIC and BIC (Table 7 in Appendix), quantile–quantile plots and Kolmogorov–Smirnov test (Fig. 6 in Appendix and Table 8 in Appendix) a gamma-distribution with identity link seemed most appropriate when modelling costs for ICU patients. The coefficients of the estimated GLMs can be found in Table 4. Higher Elixhauser comorbidity scores, longer LOS on the general ward, ICU without mechanical ventilation and ICU with mechanical ventilation and the presence of a complication were significantly positively associated with the total per patient hospitalization costs. There were no covariates significantly negatively associated with the total per patient hospitalization. None of the estimated coefficients were affected by multicollinearity.

**Table 4:** Estimated GLM with Gamma error distribution and identity link function for ICU patients

Coefficient (standard error)	Full sample	Outliers removed
Constant	4.61 (289.83)	211.66 (251.34)
Age	-4.24 (4.43)	-2.69 (3.78)
Gender female	-195.12 (206.86)	-299.66 (177.01)
Mortality	363.99 (206.90)	468.38 (186.53)*
Elixhauser comorbidity score	130.60 (56.09)*	-43.39 (53.04)
General ward (days)	414.20 (22.17)***	421.34 (19.21)***
ICU non-mechanical ventilation (days)	927.45 (45.52)***	909.33 (39.41)***
Mechanical ventilation duration (days)	2224.84 (70.24)***	2174.18 (59.54)***
ECMO duration (days)	350.62 (191.73)	440.65 (167.92)**
Dialysis (yes/no)	343.59 (320.11)	287.01 (283.49)
Cardiopulmonary resuscitation	-282.52 (292.87)	-86.80 (251.40)
Complication	2554.40 (1122.82)*	2837.93 (993.46)**
Number of samples	510	506
Weighted sample size	350.57	372.31
*P* value Kolmogorov–Smirnov test	0.04*	0.83
*P* value dispersion test	< 0.001***	< 0.001***
*P* value outlier test	0.02*	0.08

^*^ *p* < 0.05; **p < 0.01; ***p < 0.001

After sensitivity analysis none of the estimated coefficients changed majorly. However, mortality and ECMO were positively significantly associated with the total per patient hospitalization costs after deletion of the outliers. The Elixhauser comorbidity score was no longer significant. The estimated GLM without outliers can be found in Table 4.

## Discussion

### Main findings

While COVID-19 hospitalization costs are essential for policymakers to make informed health care decisions, to date not much is known about these costs in western European countries. This is, to our knowledge, the first study examining the costs of COVID-19 hospitalizations in Germany. To explore hospitalization costs of COVID-19 non-ICU and ICU patients in Germany we analyzed administrative data from the University Hospital Frankfurt am Main. Median total hospitalization costs per non-ICU patients and per ICU patients, without and with mechanical ventilation were respectively € 3,010 (range: € 684—€ 45,803, Q1 – Q3: € 2,224—€ 5,273), € 5,887 (range: € 705—€ 38,028,Q1– Q3: € 3,054—€ 10,879) and € 21,536 (range: € 724—€ 209,814, Q1 – Q3: € 7,504—€ 43,480). Over time total per patient hospitalization costs increased slightly for non-ICU patients, while they decreased for ICU patients.

Next, Propensity scores were estimated for length of stay (LOS) at the general ward and mechanical ventilation (MV) duration, using CBPS for continuous treatment. Costs for each additional day in the general ward and each additional day in the ICU with and without MV were estimated by regressing the total per patient hospitalization costs on the LOS and the presence or absence of several treatments using GLM, while controlling for patient characteristics, comorbidities, and complications. Total hospitalization costs per non-ICU patient were significantly positively associated with mortalities, higher Elixhauser comorbidity scores and longer LOS on the general ward. Age and cardiopulmonary resuscitation were significantly negatively associated with the total hospitalization costs per non-ICU patient. However, as Elixhauser comorbidity score and age were included in the CBPS and were therefore balanced before inclusion in the GLM, their estimated coefficients were presumably biased. Each additional day on the general ward for non-ICU patients was found to cost on average € 463.66 (SE: 15.89). For ICU patients higher Elixhauser comorbidity scores, longer LOS on the general ward, longer ICU LOS without mechanical ventilation, longer duration of mechanical ventilation and the presence of a complication showed a significant positive relationship with the total per patient hospitalization costs. However, since the Elixhauser comorbidity score was included in the CBPS its coefficient was presumably biased. This also holds for the estimated coefficients of age and gender. There were no covariates significantly negatively associated with the total hospitalization costs per ICU patient. Additional days on the general ward, non-mechanically ventilated days in the ICU, mechanically ventilated days in the ICU and days of ECMO, were estimated at respectively € 414.20 (SE: 22.17), € 927.45 (SE: 45.52), € 2224.84 (SE: 70.24) and € 350.62 (SE: 191.73).

### Relationship to other hospitalization costs studies

Only two other studies examined the costs of COVID-19 hospitalizations in western Europe. Carrera-Hueso et al. (2021) estimated total hospitalization costs from a hospital perspective in Spain, respectively per non-ICU patient and per ICU patient at € 60,997 and € 341,845 (adjusted to German 2021 euros, using harmonised indices of consumer prices and purchasing power parities from Eurostat [22, 49], rounded to whole euros) [17]. Moreover, in a study including six public health hospitals in Italy total per patient hospitalization costs from a hospital perspective were € 6,668, € 9,188 and € 18, 275 (inflated to German 2021 euros [22, 49], rounded to whole euros), for respectively low-complexity care, medium-complexity care and high complexity care of COVID-19 patients [50]. However, considering the different health-care systems between different countries we consider these cost to be incomparable. Furthermore, the previously mentioned study by Goettler et al. (2022) examined the costs of influenza hospitalizations in Germany in a sample with non-ICU and ICU patients and found total median costs per patient and per admission to be € 1,858 (inflated to 2021 [22], rounded to whole euros) [29]. These costs are evidently lower than the total COVID-19 hospitalization costs in our study. However, of note is that the economic perspective taken of these studies were different (i.e. hospital perspective vs. German statutory health insurance perspective).

In our study we found a slight positive trend for non-ICU patients and a negative trend for ICU patients in total per patient COVID-19 hospitalization costs over time, indicating that total per patient hospitalization costs early in the pandemic might not be appropriate later on. As far as we are aware, no study examined the total per patient hospitalization costs of COVID-19 patients in western Europe over time. However, a large study including 247,590 hospitalized COVID-19 patients in the United States of America showed decreasing total per patient hospitalization costs over time for both non-ICU and ICU patients [32]. A possible explanation for the different results may lie in the different health care systems.

Only one other study looked at daily hospitalization costs in western Europe. Foglia et al. (2022) estimated daily hospitalization costs from a hospital perspective at € 546, € 804 and € 1610 (inflated to German 2021 euros [22, 49], rounded to whole euros), for respectively low-complexity care, medium-complexity care and high complexity care using data from six public health hospitals in Italy [50]. Other researchers estimated the costs of a day in the ICU in Germany for non-COVID-19 patients between €744 and €1,462 (inflated to 2021 [22], rounded to whole euros) [23–28]. However, all of these studies analyzed the costs from a hospital perspective. The estimated costs for each additional day in the ICU for COVID-19 patients without mechanical ventilation were in accordance with these estimated amounts. Yet, our research showed that the costs for an extra day of mechanical ventilation for COVID-19 patients are more than twice the costs for an additional day in the ICU for non-mechanically ventilated COVID-19 patients. This partly is in agreement with earlier research for non-COVID-19 ICU patients, which showed that mechanical ventilation is a main driver for increased costs of patient care [26, 51–53]. However, the estimated daily costs of a COVID-19 patient with mechanical ventilation were higher compared to estimated costs for non-COVID-19 mechanically ventilated ICU patients in Germany, both taken from a hospital perspective [25, 26]. In addition, ECMO costs were estimated at € 350.62 (SE: 191.73) per day. These costs were relatively low. Since COVID-19 patients in the ICU are in general severely ill and already have high costs it could be that the use of ECMO did not trigger a higher reimbursement in the case of the COVID-19 related DRG codes. In addition, the extrabudgetary compensation for ECMO was not included in the total per patient hospitalization costs. In Germany, there is a high use of ECMO therapy, possibly caused by this extrabudgetary compensation [54, 55]. The amount of this extrabudgetary compensation depends on the patients’ characteristics and the severity of the illness and is negotiated individually by each hospital and can vary from € 600 to ten thousands of euros per hospital [56]. In addition, we note that the estimates for the costs of the duration of ECMO had a high standard error, showing quite some uncertainty.

We found that for non-ICU patients cardiopulmonary resuscitation (CPR) had a significant negative effect on the costs, while the costs for deceased patients were significantly higher compared to patients who were discharged. However, the vast majority of the patients in the non-ICU sample receiving CPR were deceased. Moreover, in the non-ICU sample the number of patients that received CPR was extremely low. Therefore, even though those costs were significantly lower for these patients, the generalizability of the estimated costs for CPR is most likely poor. No interaction effect between mortality and CPR was added as this interaction would have been highly colinear with the CPR covariate. Next, the presence or absence of dialysis did not make a significant difference for the costs of either non-ICU COVID-19 patients nor ICU COVID-19 patients. The total per patient hospitalization costs used in this study excluded the extrabudgetary compensation. This might cause the estimated costs for dialysis to be lower than expected. Moreover, the estimated standard errors were relatively large. An explanation for this could be that the dialysis variable included all diverse types of dialysis. The most common types of dialysis in Germany for COVID-19 patients include intermittent haemodialysis and continuous, venovenous, pump-driven haemodialysis [57]. The variation in these dialysis types and the lack of the duration of dialysis in our data could have led to non-significant estimated costs for dialysis. In addition, for the non-ICU COVID-19 patients, the sample contained a small number of patients receiving dialysis. Therefore, the non-ICU COVID-19 sample could have been too limited to provide a narrow confidence interval of the costs for dialysis.

### Strengths and limitations

An extensive study was performed into the costs of COVID-19 patients in Germany. Overall, the cost estimates provide a clear overview of the hospitalization costs for COVID-19 patients in Germany. Cost estimates were based on samples without missing values. Models were fitted doubly robust, that is, controlling for confounding with outcome regression and propensity score weighting. Moreover, sensitivity analysis was performed, and all estimated models were robust against outlier removal. As expected, the estimated coefficient for an additional day in the general ward for the non-ICU patients was relatively close to the estimated coefficient for ICU patients in the general ward. Furthermore, the error distribution of the GLMs for the ICU patients showed reasonable statistical fit. Finally, the estimations of daily costs enable these results to be used in various stages of the pandemic and endemic as it offers the flexibility to estimate total per hospitalization costs for different LOS.

Our research was subject to several limitations. Firstly, the administrative data in this study was from a single hospital in Germany. In the present study we applied a flat base rate, which is the basis rate for all hospitals in Germany before negotiation. However, the patient population in other hospitals can be different. Ideally, the same modelling approach would be applied to administrative data from other (university) hospitals in Germany. Relatedly, the extrabudgetary compensation was not included in the analysis, these rates are negotiable and therefore not disclosed. Our estimated costs together with the extrabudgetary compensation will fully reflect the costs of COVID-19 hospitalizations from a German statutory health insurance perspective. Nevertheless, the inclusion of these negotiable rates will impact the generalizability of this study as these rates can differ substantially between different hospitals [56]. Moreover, in our study costs were not indexed to the same year since the price history of DRGs and other administrative prices do not follow the general consumer price index. However, correcting the costs to the same index year will lead to only minor differences. Next, as we are potentially moving towards a post-pandemic situation the patient population might change. However, we controlled for patient characteristics, comorbidities, and complications. Therefore, we expect that these estimated daily costs are generalizable, also after the pandemic. However, the generalizability of the results can be influenced by major changes in the treatment of COVID-19, changes in the population immune response due to vaccinations and infections, and antigenic drift of SARS-CoV-2. In addition, administrative costing data can be subjective to mistakes. Demographics that are not directly relevant for the costs like mild obesity are occasionally underreported [58]. Moreover, administrative costing data do not necessarily reflect the actual costs in a one-to-one way [59]. Reimbursed costs can potentially be lower or higher than the actual costs [60].

All the estimated models suffered from underdispersion. However, note that underdispersion is in this case not problematic since it leads to conservative standard errors, i.e., larger confidence intervals [61]. Hence, despite that the estimates are not the most efficient estimates they do give a reasonable impression of the effect on the hospitalization costs. Furthermore, the best fitted GLM for the non-ICU patients had a relatively poor fit. Therefore, the estimated costs for non-ICU patients need to be interpreted with caution. In addition, it would be interesting to compare our estimated costs for non-ICU patients to the costs estimated by the InEK. However, unfortunately these data were not available at the time the current study was performed. Moreover, while we were able to estimate the effect of an additional day in the hospital on the total per patient hospitalization costs, the best fitted model assumes that these costs are constant over time. In reality, the first day of hospitalization is known to be the most expensive. For instance, Rapoport et al. (2003) showed that the first day in the ICU is more than 1.5 times as expensive as later days in the ICU [62]. This effect was not visible in our fitted model. Relatedly, the estimated coefficients for the general ward, ICU and mechanical ventilation duration cannot directly be interpreted as the costs for a day in the hospital as this disregard the effect of age, gender, and comorbidities on the costs. These estimates can rather be interpreted as the effect of one extra day in the general ward or ICU on the total per patient hospitalization costs.

## Conclusion

This study is, as far as we are aware, the first study examining COVID-19 hospitalization costs in Germany. Total per patient hospitalization costs and daily hospitalization costs were extensively studied. Our study showed that using total per patient influenza hospitalization costs as a proxy for total per patient COVID-19 hospitalization costs is potentially inadequate. Moreover, total German hospitalization costs observed early in the pandemic might not be representative for the situation later on in the pandemic. Overall, estimated daily costs were in agreement with daily costs found in literature for non-COVID-19 patients, except for higher estimated costs for mechanical ventilation. Considering the increased costs for mechanical ventilation it is recommended from a health-economic perspective to prevent mechanical ventilation by for example early interventions. The total per patient hospitalization costs and estimated costs for an extra hospitalization day in this study can be used to estimate the budget impact by COVID-19 social restrictions or as input parameters for economic models, such as for cost effectiveness studies of COVID-19 vaccinations or novel COVID-19 therapies. This can potentially improve the precision of COVID-19 cost effectiveness studies in Germany and will allow health care policymakers to provide better informed health care resource decisions in the future.

## Data Availability

The datasets analyzed during the current study are not publicly available due to privacy of the patients involved, but are available from the corresponding author on reasonable request. The R codes of the models are openly available in Github at https://github.com/UMCG-Global-Health/german_COVID-19_costs.

## Appendix

**Table 5:** AIC and BIC for estimated GLM’s with different error distributions and link functions for non-ICU patients

Family	Link function	AIC	BIC
Gaussian	Identity	10,516.52	10,560.46
Gaussian	Log	10,614.11	10,658.05
Gamma	Identity	36.72	80.66
Gamma	Log	37.09	81.03
Inverse Gaussian	Identity	36.82	80.76
Inverse Gaussian	Log	37.15	81.09

**Table 6:** Results of Kolmogorov–Smirnov test, dispersion test and outlier test for the gamma and inverse gaussian error distribution with an identity and log link function for the non-ICU patients

	Gamma (identity)	Gamma (log)	Inverse Gaussian (identity)	Inverse Gaussian (log)
Kolmogorov–Smirnov test (*p*-value)	< 0.001***	< 0.001***	< 0.001***	< 0.001***
Dispersion test (*p*-value)	< 0.001***	0.14	< 0.001***	0.80
Outlier test (*p*-value)	0.033*	< 0.001***	0.033*	0.008**

^*^ *p* < 0.05; ***p* < 0.01; ****p* < 0.001

**Table 7:** AIC and BIC for estimated GLM with different error distributions and link functions for ICU patients

Family	Link function	AIC	BIC
Gaussian	Identity	10,535.43	10,590.48
Gaussian	Log	11,479.45	11,534.49
Gamma	Identity	45.80	100.85
Gamma	Log	47.01	102.05
Inverse Gaussian	Identity	46.63	101.68
Inverse Gaussian	Log	47.07	102.12

**Table 8:** Results of Kolmogorov–Smirnov test, dispersion test and outlier test for the gamma and inverse Gaussian error distribution with an identity and log link function for the ICU patients

	Gamma (identity)	Gamma (log)	Inverse Gaussian (identity)	Inverse Gaussian (log)
Kolmogorov–Smirnov test (*p*-value)	0.040*	0.009**	< 0.001***	< 0.001***
Dispersion test (*p*-value)	< 0.001***	0.12	< 0.001***	0.77
Outlier test (*p*-value)	0.020*	0.004**	0.003**	< 0.001***

^*^ *p* < 0.05; ***p* < 0.01; ****p* < 0.001

## Abbreviations

AIC: Akaike Information Criterion
BIC: Bayesian Information Criterion
CBPS: Covariate balancing propensity score
CPR: Cardiopulmonary resuscitation
DRG: Diagnosis related groups
ECMO: Extracorporeal membrane oxygenation
GLM: Generalized linear model
ICD-10: International Statistical Classification of Diseases and Related Health Problems edition 10
ICU: Intensive Care Unit
InEK: Institute for the Hospital Remuneration System
IQR: Interquartile range
MV: Mechanical ventilation
LOS: Length of stay
Q1: First quartile
Q3: Third quartile
Q-q- plots: Quantile–quantile plots
rt-PCR: Reverse transcription Polymerase chain reaction
Sd: Standard deviation
SE: Standard error
VIF: Variance Inflation Factor
